# Prognostic Factors for Visual Outcome in Traumatic Cataract Patients

**DOI:** 10.1155/2016/1748583

**Published:** 2016-08-09

**Authors:** Ying Qi, Yan F. Zhang, Yu Zhu, Ming G. Wan, Shan S. Du, Zhen Z. Yue

**Affiliations:** Department of Ophthalmology, The First Affiliated Hospital of Zhengzhou University, Zhengzhou 450052, China

## Abstract

*Purpose*. To investigate the prognostic factors for visual outcome in traumatic cataract patients.* Methods*. The demographic features of traumatic cataract patients in Central China were studied. The factors that might influence the visual outcome were analyzed. The sensitivity and specificity of OTS (ocular trauma score) in predicting VA were calculated.* Results*. The study enrolled 480 cases. 65.5% of patients achieved VA at >20/60. The factors associated with the final VA were initial VA, injury type, wound location, the way of cataract removal, and IOL implantation. The sensitivities of OTS in predicting the VA at NLP (nonlight perception), LP/HM (light perception/hand motion), and ≥20/40 were 100%. The specificity of OTS to predict the final VA at 1/200-19/200 and 20/200-20/50 was 100%.* Conclusion*. The prognostic factors were initial VA, injury type, wound location, cataract removal procedure, and the way of IOL implantation. The OTS has good sensitivity and specificity in predicting visual outcome in traumatic cataract patients in long follow-up.

## 1. Introduction 

Ocular trauma has been a common reason of visual morbidity, which causes heavy psychological and economic burden to victims and society [1–3]. In the United States, there are approximately 2.5 million cases of eye trauma every year [4]. Ocular trauma includes mechanical eye injury (open globe injury and closed globe injury) and nonmechanical eye injury. Each of these categories can cause traumatic cataract, which might damage the vision [5].

The cause of traumatic cataract is complex. It may have been caused by the rupture of the lens capsule or zonular ligament, the disorder of lens metabolism, or the oscillation of lens cortex caused by collision. The occurrence of traumatic cataract could be observed immediately or several years after eye injury. The shape of traumatic cataract may be total, local, rosette, or swollen or other irregular shapes [6]. The location of the opacity may be at anterior or posterior cortex or the capsule. Any of these types of cataract will cause visual problems to the patients. How to predict the visual outcome of traumatic cataract has not been well documented.

The OTS (ocular trauma score) has been developed by Kuhn et al.'s study group that aims to predict the visual outcome of traumatic eyes [7], which has showed a predictable value for different types of ocular trauma [8–10]. And recently some researchers have reported that the OTS can indicate the final vision for traumatic cataract patients [11, 12]. Has OTS also been effective for traumatic cataract cases in Central China? To answer this question, we retrospectively reviewed the medical records of traumatic cataract in our hospital in the last five years and analyzed the data. The details were delineated as follows.

## 2. Methods and Patients

Our research has been approved by the Medical Ethical Committee of our university. The written or oral consent has been obtained from all participants or the guardians.

The medical records of traumatic cataract patients who received cataract surgery in our Ophthalmic Center from 2010 to 2014 were reviewed. The retrieved information included age, gender, profession, causative agent, type of injury, initial visual acuity (VA) after injury, diagnosis, treatment, and final VA (the best corrected VA at the last follow-up).

The diagnosis and classification of ocular trauma in this paper are based on the Birmingham Eye Trauma Terminology (BETT) which is a standardized ocular trauma definition and classification system for mechanical eye injuries [13–15]. The eye wound location was defined by the Ocular Trauma Classification Group: zone I, injuries limited to the cornea; zone II, injuries confined to the anterior 5 mm of the sclera; and zone III, injuries involving more than 5 mm posteriorly from the limbus [13].

The causative agents were divided into sharp metal items (knife, scissors, forks, etc.), blunt injury (fist, stone, toy, etc.), animal (bite, hit, strip, etc.), electrical injury, chemical injury, and others.

Based on the injury condition, the cataract removal was performed in four different ways: phacoemulsification, extracapsular cataract extraction (ECCE) not phacoemulsification, intracapsular extraction, or lensectomy. The intraocular lens (IOL) might be implanted in the capsular bag, in the anterior chamber (iris-clipped or placed on the lens capsule), or fixed in the ciliary sulcus. The IOL may be implanted at the first stage or second stage. During the surgery, the combined procedures include wound closure, tissue repair, foreign body removal, or vitreous or retinal operation.

After operation, topical corticosteroid and antibiotics were applied. If the eyes showed infectious signs, the antibiotics were applied locally and systemically. The patients were followed up at 1 week, 1 month, 6 months, and 12 months postoperatively. For the enrolled patients, the follow-up was at least 6 months. At each follow-up, the corrected VA was recorded. The eyes were examined by slit-lamp microscope and direct ophthalmoscope. Sometimes, an additional examination, like B-ultrasonography, CT, or MRI, was needed. The posterior capsular opacity was observed in some patients during follow-up, which could be treated by capsulotomy with laser or scissors.

The ocular trauma score (OTS) was first introduced by Kuhn et al. in 2002 [7], which is a simplified system for standardized assessment and visual prognosis associated with eye injury. The certain numerical values were classified as OTS variables (Table 1). The summation of the variables of each category indicated the possible final vision.

The raw points of variables of each patient were calculated, summated, and categorized to different groups. Then, the potential VA of five categories was calculated. The potential VA was compared with the actual VA; then, the sensitivity and the specificity of OTS in each category were calculated.

The data were analyzed with SPSS software, version 17.0 (SPSS, Inc., Chicago, IL, USA). Descriptive statistics and cross tabulation were used to compare the effects of different variables. The dependent variable was VA > 20/60. The correlation of initial and final VA was analyzed by Spearman Correlation. All values in our study were two-sided, and a value of less than 0.05 was considered statistically significant.

## 3. Results

### 3.1. Demographic Data

This study has reviewed the medical data of 480 cases of traumatic cataract from 2011 to 2014. There were 324 males and 156 females (*x* ^2^ = 117.6, *P* < 0.01). The ratio of males to females is 2.1. The age of patients ranged from 2 months to 83 years with an average of 41.5 ± 19.3 years. The average age is 40.9 ± 17.7 for males and 42.1 ± 16.8 for females. There were 98 cases (20.4%) under 14 years old and 41 cases (8.5%) under 5 years old. The average follow-up was 11.2 ± 2.5 months.

The professions were 191 peasants (39.8%), 165 workers (34.4%), 78 students (16.3%), and 46 cases (9.6%) of other professions (*x* ^2^ = 159.0, *P* < 0.01) in our study. The causative agents included sharp agents (324 cases, 67.5%), blunt agents (82 cases, 17.1%), animal (29 cases, 6.0%), electricity (12 cases, 2.5%), chemicals (28 cases, 5.8%), and others (5 cases, 1.0%) (*x* ^2^ = 1126.4, *P* < 0.01).

There were 437 cases (91.0%) of mechanical injury and 43 cases (9.0%) nonmechanical injuries (*x* ^2^ = 646.8, *P* < 0.01). In mechanical injury, there were open globe injury (354 cases, 73.8%) and closed globe injury (83 cases, 17.3%) (*x* ^2^ = 336.1, *P* < 0.01). Among the open globe injuries, there were 255 cases (72.0%), 74 cases (20.9%), and 25 cases (7.1%) with wound at zone I, zone II, and zone III (*x* ^2^ = 373.1, *P* < 0.01). In nonmechanical injuries, there were chemical injury (28 cases, 5.8%), electrical injury (12 cases, 2.5%), and other types (3 cases, 0.6%) (*x* ^2^ = 33.6, *P* < 0.01).

Four different cataract removal procedures were performed on the patients. The cataract phacoemulsification was performed in 62 cases (12.9%), ECCE was performed on 247 eyes (51.5%), cataract intracapsular extraction was performed on 123 eyes (25.6%), and lensectomy was performed on 48 eyes (10.0%) (*x* ^2^ = 274.3, *P* < 0.01).

The IOL was implanted in the capsule (177 eyes, 36.9%) and in the ciliary sulcus (242 eyes, 50.4%) or clipped at the iris (32 eyes, 6.7%) (*x* ^2^ = 379.5, *P* < 0.01). There was no IOL implantation in 29 cases (6.0%).

Except for cataract operation, some other procedures were performed. There were wound closure (356 eyes, 74.2%), pupillary formation (95 eyes, 19.8%), antiglaucoma surgery (87 eyes, 18.1%), vitreous surgery (56 eyes, 11.7%), retinal surgery (49 eyes, 10.2%), and others (92 eyes, 19.2%).

### 3.2. Visual Outcomes of Traumatic Cataract Patients

The initial VA and final VA of traumatic cataract were shown in Table 2. There were 75 eyes (16.2%) with initial VA of >20/60 and 305 eyes (65.6%) with final VA of >20/60 (*x* ^2^ = 235.0, *P* < 0.01). The initial VA was positively associated with final VA (*r* = 0.93, *P* < 0.01).

For different professions, the final VA of >20/60 was in 122 eyes (64.2%) in peasants, 110 eyes (67.1%) in workers, 55 eyes (66.2%) and 51 eyes (66.2%) in students, and 22 eyes (62.9%) in other professions (*x* ^2^ = 0.45, *P* = 0.93). The final VA of different types of injuries was shown in Table 3. The final visual outcomes among different types were significantly different (*x* ^2^ = 27.6, *P* < 0.01). The final VA of closed globe injury was better than that of open globe injury (*x* ^2^ = 10.1, *P* < 0.01). In open globe injury, the final VA of >20/60 was found in 128 eyes (37.5%) in zone I, 26 eyes (7.6%) in zone II, and 2 eyes (0.6%) in zone III. The eyes with wound in zone I had better vision (*x* ^2^ = 22.7, *P* < 0.01) than in other zones.

The final VA with different cataract removal procedures was shown in Table 4. The patients with phacoemulsification had better VA than those with other procedures (*x* ^2^ = 92.3, *P* < 0.01). The results of final vision with different IOL implantation procedures were shown in Table 5.

### 3.3. Prognostic Value of OTS

The actual and calculated OTS of each patient were summated and analyzed. And the sensitivity and specificity were calculated and shown in Table 6. The sensitivities of OTS predicting the VA at NLP, LP/HM, and ≥20/40 were 100%. The specificity to predict the final VA at 1/200–19/200 and 20/200–20/50 was 100%.

## 4. Discussion

This study has three parts: clinical features of traumatic cataract patients in Central China, factors associated with visual outcome, and predictive value of OTS.

The average age in our study was 41.5 ± 19.3 years. Shah et al. reported that the average age was 27 years [6, 11] in India and Serna-Ojeda et al. reported that it was 46 years in Mexico [16]. In our study, the ratio of males to females is about 2.1 : 1, different from other reports with ratio 3 : 1 [16, 17].

Open globe injury accounted for 73.8% in traumatic cataract in our study. In other researches, these percentages vary from 65.2% to 80% [6, 11, 16, 18, 19]. In our study, only 12.9% underwent phacoemulsification, which is much lower than the 96.3% reported by other researches [16]. Some patients were chosen to do the ECCE, not phacoemulsification in our city. This is because of the economic reason, to save money for patients.

In our study, IOL implantation was performed in 94% of patients. In other researches, about 80% of patients accepted IOL implantation [11]. In our results, 18.1% received combined cataract and antiglaucoma surgery. Similar to Rogers et al.'s report [20], 28% of the patients had hypertension complications after traumatic cataract.

In our present study, 65.5% of the eyes achieved a final VA of >20/60 and 49.8% of the eyes obtained VA of ≥20/40. Many other articles have also reported the final VA in traumatic cataract patients. Serna-Ojeda et al. reported that 58.7% of cases obtained VA ≥ 20/40 [16]. Rogers et al. reported 66.7% of cases obtained VA of >20/60 [20]. Memon et al. reported that 70.8% of cases obtained VA of >20/60 [17]. Shah et al. reported that 54.3% of cases obtained VA of >20/60 [18] and 31% of cases obtained VA of ≥20/40 [11]. Bekibele and Fasina reported that 35.6% of cases obtained VA of >20/60 [21].

After treatment, the final VA was much better than the initial VA statistically in our results. The same results have been reported by other authors [6, 22–24]. Treatment for traumatic cataract is different from senile cataract although most cataract procedure steps were similar. First, the wound should be treated before the cataract in open globe injury, like eyelid, corneal or scleral wound, and lacrimal apparatus injury. Second, the traumatic cataract operation always is more complicated than the traditional cataract operation. In some cases, the patients may have broken anterior capsule, which makes complete capsulorhexis very difficult. Some cases are complicated with zonular rupture or lens luxation. The posterior capsular rupture was much more common in traumatic cataract surgery than in conventional cataract operation. So, sometimes lensectomy or vitrectomy is necessary. As Kuhn reported, traumatic cataract procedure is an individualized, consciously made decision regarding what to do and when and how to do it to achieve the best possible outcomes [24].

The possible factors which may influence the final VA were initial VA, injury type, wound location, cataract removal procedure, and the way of IOL implantation. (1) The initial VA was positively associated with the final VA. The patients with better initial VA usually had better final VA. The initial VA was also the predictor for visual outcome in open and closed globe injuries [1, 2]. (2) The final VA of open globe injury was better than that of closed globe injury. Shah et al. had a similar conclusion in their study [18]. (3) The location of the wound in open globe injury will influence the vision. The VA of zone I was higher than that of zone II. The VA of zone II was higher than that of zone III. This is because the retina is located at the posterior part of the eyeball, which is the vital part for visual message transition. (4) The patients who underwent different cataract removal procedures had different visual outcomes. Phacoemulsification usually needed better operational conditions, like stable anterior chamber, complete capsular bag, and sufficiently strong suspensory ligament. For patients with broken capsule, ECCE or other methods may be applied. (5) The IOL implantation in different ways had different results. IOL implanted in the capsule is the ideal position for vision. For patients without complete or enough capsules, the IOL was fixed in the ciliary sulcus or the iris.

The OTS was used in many ocular traumas to predict the vision. Sobaci et al. reported that OTS may provide prognostic information in deadly weapon-related open globe injuries [25]. Unal et al. reported that OTS provided reliable information about the prognosis of deadly weapon-related open globe injuries with intraocular foreign bodies [26]. Uysal et al. reported that OTS might provide prognostic information in children with open globe injuries [27]. OTS also had a good predictive value in firework-related eye injuries [9] and closed globe injuries [28].

About the predictive value of OTS on traumatic cataract in children, Zhu et al. have reported that OTS has a high ability to predict visual outcome for pediatric traumatic cataract following penetrating ocular trauma [12]. Shah et al. also reported that OTS was a reliable predictor of the final vision of pediatric traumatic cataract [29]. There was only one reference that mentioned the value in traumatic cataract in adults and children. Shah reported that OTS was found as a reliable tool to predict visual outcome in traumatic cataracts 6 weeks postoperatively [11]. Our paper has observed the long term of final VA of cases to evaluate the predictive value of OTS for traumatic cataract patients in long-term follow-up. And the OTS had high predictive sensitivity and high specificity for traumatic cataract patients in the long term.

In conclusion, our paper has found five factors influencing the final VA: initial VA, injury type, wound location, cataract removal procedure, and IOL implantation method. The OTS has high sensitivity and specificity for predicting visual outcome of traumatic cataract patients in long-term follow-up.

## Figures and Tables

**Table 1:** The variables and raw points in OTS.

Variables	Raw points
Initial vision	
NLP	60
LP/HM	70
1/200–19/200	80
20/200–20/50	90
≥20/40	100
Rupture	−23
Endophthalmitis	−17
Perforating or penetrating injury	−14
Retinal detachment	−11
Afferent pupillary defect	−10

NLP: nonlight perception; LP: light perception; HM: hand motion.

**Table 2:** The initial and final VA.

	Initial VA		Final VA	
	Frequency	Percentage (%)	Frequency	Percentage (%)
NLP	18	3.8	15	3.1
LP/HM	171	35.6	33	6.9
1/200–19/200	130	27.1	48	10.0
20/200–20/50	123	25.6	131	27.3
≥20/40	24	5.0	239	49.8
Uncooperative	14	2.9	14	2.9

Sum	480	100.0	480	100.0

The initial VA and final VA of traumatic cataract patients at different levels were shown in the table. NLP: nonlight perception; LP: light perception; HM: hand motion.

**Table 3:** The final VA of different types of injuries.

	≤20/60		>20/60	
	Frequency	Percentage (%)	Frequency	Percentage (%)
Open globe injury	125	26.8	216	46.4
Closed globe injury	15	3.2	67	14.4
Chemical injury	19	4.1	9	1.9
Electrical injury	1	0.2	11	2.4
Others	1	0.2	2	0.4

Sum	161	34.5	305	65.5

The final VA of different injury types related to traumatic cataract was shown in the table.

**Table 4:** Final VA of different cataract removal procedures.

Procedure	≤20/60		>20/60	
	Frequency	Percentage	Frequency	Percentage
Phacoemulsification	9	1.9	53	11.4
Extracapsular cataract extraction	47	10.1	189	40.6
Intracapsular cataract extraction	73	15.7	48	10.3
Lensectomy	32	6.9	15	3.2

Sum	161	34.5	305	65.5

The final VA of different traumatic removal procedures was shown in the table.

**Table 5:** Final VA of patients with four different IOL implantation procedures.

	≤20/60		>20/60	
	Frequency	Percentage (%)	Frequency	Percentage (%)
In the capsule	34	7.3	143	30.7
In the ciliary sulcus	78	16.7	152	32.6
On the iris	22	4.7	10	2.1
No implantation	27	5.8	0	0.0

Sum	161	34.5	305	65.5

The frequency and percentage of VA in patients with four different traumatic cataract removal procedures were shown in the table.

**Table 6:** The sensitivity and specificity of OTS in predicting the final VA.

	NLP	LP/HM	1/200–19/200	20/200–20/50	≥20/40
Sensitivity (%)	100.0	100.0	92.9	90.2	100.0
Specificity (%)	99.1	99.4	100.0	100.0	94.8
Positive value (%)	78.5	93.0	100.0	100.0	95.3
Negative value (%)	100.0	100.0	99.2	96.3	100.0
